# Empowering vision: the impact of nursing-led educational program on patients with dry eye syndrome

**DOI:** 10.1186/s12912-024-02318-9

**Published:** 2024-09-27

**Authors:** Emad Abd El Gawad Ali Rabie, Jehan Y. ElRazkey, Heba Abdelmowla Ahmed

**Affiliations:** 1 Department of Medical-Surgical Nursing, Faculty of Nursing, Alexandria University, Alexandria, Egypt; 2 Department of Medical-Surgical Nursing, Faculty of Nursing, The British University in Egypt, Cairo, Egypt

**Keywords:** Dry Eye Syndrome, Educational Program, Health-related Outcomes, Vision, Ocular Symptoms

## Abstract

**Background:**

Dry eye syndrome (DES) is a widespread ocular condition affecting the general population. It is a complex disorder affecting the eye surface, characterized by a tear film imbalance and ocular symptoms such as eye ache, burning, irritation, dryness, blurred vision, and foreign body sensation. DES can reduce visual acuity, increase the risk of ocular infection, and significantly impact daily activities and quality of life.

**Aim:**

Determine the impact of nursing-led educational program on the management of DES and patients’ health outcomes including the intensity of DES symptoms and their influence on visual-related functions.

**Methods:**

The study was conducted with a quasi-experimental design. Sixty adult patients diagnosed with DES were selected using a convenience sampling method. Two tools were employed for the collection of data. Tool I: Precipitating factors of DES structured interview schedule. Tool II: Ocular surface disease index (OSDI) to assess ocular irritation symptoms associated with DES and their effect on functions related to vision.

**Results:**

A statistically significant decrease in the mean scores of OSDI in the study group two weeks after the implementation of the educational program regarding the ocular symptoms, functions associated with vision, environmental triggers, and overall score of OSDI.

**Conclusion:**

Implementing an educational program for DES is effective in relieving symptoms, boosting patients’ understanding of managing symptoms effectively, and maintaining eye health. Therefore, patients must be instructed on dry eye early detection and management to enhance health-related outcomes and self-care practices.

**Registration:**

ClinicalTrials.gov: NCT06288945.

## Introduction

Dry eye syndrome (DES) is a prevalent chronic inflammation resulting in eye discomfort, irritation, tiredness, and visual abnormalities that can make it difficult to read, use a computer, drive, or engage in other activities [1]. It represents a set of tear film disorders caused by decreased tear formation or increased tear evaporation; it causes visual symptoms, ocular surface inflammation, and discomfort. In addition, DES leads to impaired visual function and can negatively affect the outcomes of various eye surgeries including cataract surgery, glaucoma surgery, refractive surgery, and corneal transplantation [2].

In global epidemiological studies, DES can affect between 5% and 50% of the population. In the United States, 6.8% of the population (nearly 16.4 million persons) are diagnosed with DES. The prevalence of DES varies across different age groups with a higher incidence reported among older age and women tend to experience DES more frequently than men [3]. A mild case of DES can have an annual economic impact of $687, whereas a severe condition can have an annual cost of $1,267 per person. $3.8 billion was estimated to be the overall direct cost to the American economy. These expenses cover over-the-counter and prescribed medications, and punctual plug insertion [4].

Dry eye syndrome typically has multiple etiological factors [5]. It is often caused by inefficiency in tear film or lack of eyelid closure due to unconsciousness, lack of blink reflex, eyelid anomalies, heavy sedation, ocular surface disorders or surgeries, hormonal changes, and autoimmune systemic diseases [6]. Advanced age, female gender, hormonal imbalance, vitamin A deficiency, or a lack of essential fatty acids like omega-3, all raise the likelihood of developing DES. Smoking, alcohol consumption, laser-assisted in situ keratomileuses (LASIK), living in a dry atmosphere, using a computer, and wearing contact lenses also increase the risk of incidence of DES [7].

Dry eye syndrome is a frequent and significant ocular illness that requires eye care. It causes ocular symptoms such as blurred vision, ocular burning, eye ache, irritation, dryness, foreign body sensation, photophobia, visual disturbance, and difficulty in carrying out daily tasks. Furthermore, it is associated with reduced visual acuity, increased risk of ocular infection, and decreased quality of life [8, 9]. The main mechanism of DES is the instability of the tear film, which is used in addition to self-reported symptoms to diagnose DES. In the recent DES diagnosis, the quantity of tear secretion and its stability, the lipid layer thickness, and vital staining are evaluated to confirm the diagnosis [10].

Management of DES depends on the severity of symptoms and eye condition. The first line of treatment includes educational instructions about DES, adjusting the environmental factors; avoiding exposure to direct air currents, reducing screen exposure time, identifying and managing harmful local or systemic agents, applying eye lubricants, hygienic care of the eyelids, and scrubs; vitamins and omega-3 supplements [11]. The next option of treatment includes artificial tears, reversible punctual plugs, ointment application at night or moisture goggles to maintain moisture and temperature, heat pulses to clear obstructions from the meibomian glands, local anti-inflammatory drugs, and antibiotics [12].

Several researches have been executed to explore the prevalence, causes, and factors contributing to DES as well as to assess the intensity of its symptoms and appraise various diagnostic techniques and treatment modalities [13–17]. While, the crucial role of nurses in educating patients about DES and its management was not highlighted. It did not receive adequate research interest and was often overlooked. Restoring homeostasis in the ocular surface, reducing inflammation, and ensuring long-term ocular surface comfort are the main goals of DES management. Thus, this study has been executed to explore the impact of an educational nursing program on the health outcomes of patients with DES.

### This study aimed to

Identify the impact of nursing-led educational program on the management of DES and patients’ health outcomes including the severity of DE symptoms and their influence on visual-related functions.

### Research hypotheses

H_1_: Patients who receive an educational program will experience less severity of dry eye (DE) symptoms than those who do not receive the program.

H_2_: Patients who receive an educational program will experience less influence of DE symptoms on visual-related functioning compared to patients who do not receive the program.

## Materials and methods

### Materials

#### Research design and setting

This study employed a quasi-experimental research design. It was carried out at the Ophthalmology Outpatient Clinic, Main University Hospital in Alexendria, Egypt. This clinic is a primary care facility for eye-related issues, treating patients from various age groups especially adults with different ophthalmic conditions such as cataract, glaucoma, refractive errors, and DES. It operates six days a week, from Saturday to Thursday. The clinic is well-equipped with advanced diagnostic tools and provides comprehensive eye care.

### Subjects

The number of patients diagnosed with DES at the Ophthalmology Outpatient Clinic over eight months period was 110 patients, according to hospital statistics. The application G*Power Windows 3.1.9.7 was used to determine the total sample size with the subsequent criteria: Impact size was 0.25, the power was 0.85 (1-err prob), the significance level (α) was 0.05, two groups investigated, and number of measurements = 2, in accordance with Sim, and Lewis (2012) [18]. Hence a convenience sample consisting of sixty adult patients was selected. The participants were distributed into two groups, each with 30 patients: the study group and the control group. The control group was selected first based on the availability of patients and meeting the inclusion criteria; then the study group was selected to overcome selection bias. The inclusion criteria consisted of patients aged 20 to 60 years old, alert, able to communicate, and no recent ocular surgeries in the last 3 months.

**Fig. 1:**
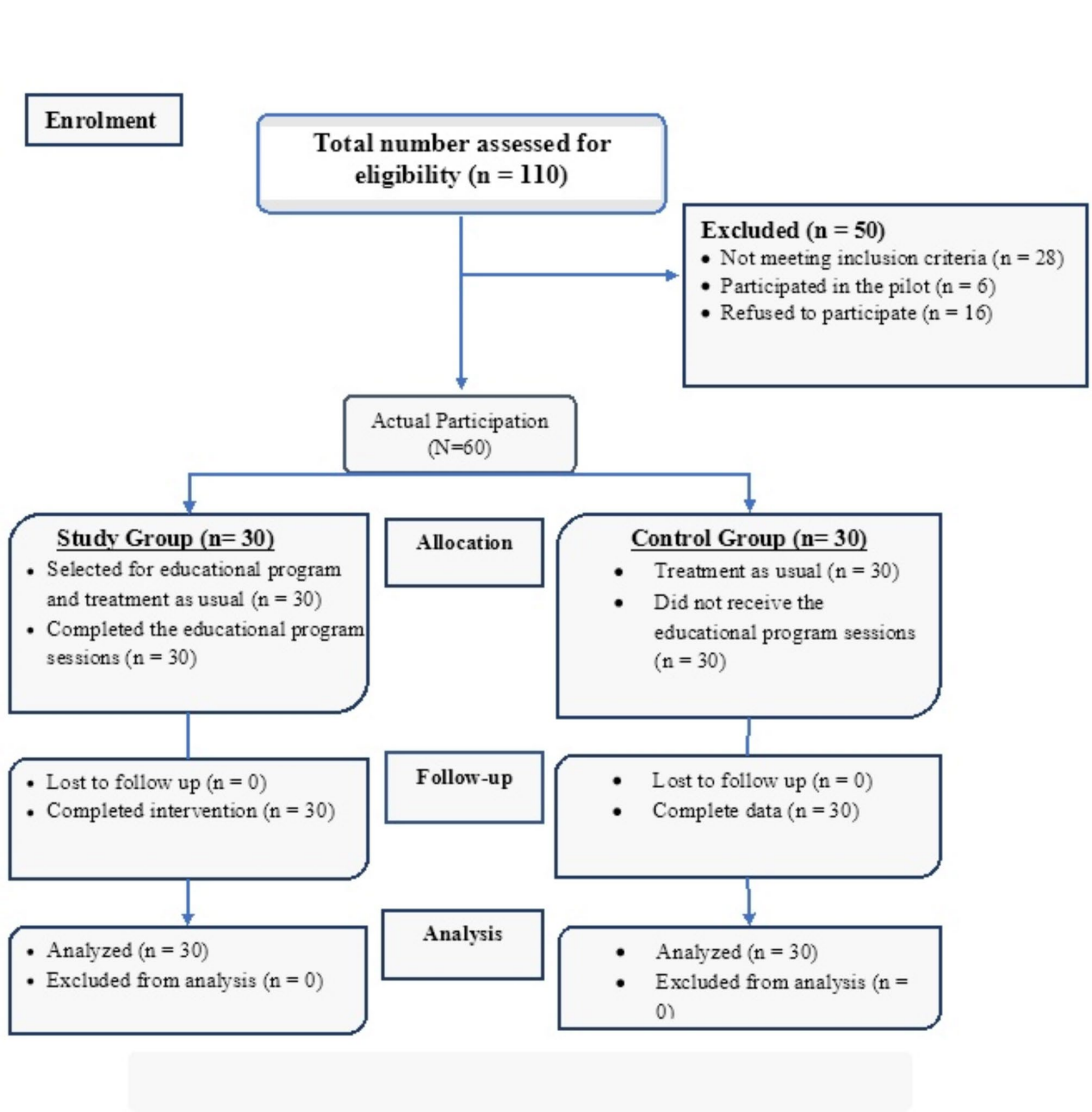
Flow chart for recruiting the study participants

The patients were chosen from the Ophthalmology Outpatient Clinic (*N* = 110) over a span of 8 months as presented in the diagram (Fig. 1). 50 patients were excluded from the total number evaluated for eligibility. This exclusion comprised 28 patients who failed to meet the required criteria, 6 patients were excluded because of their pilot study involvement, and 16 declined to be involved. All patients in the two groups received usual treatment for DES, the study group received the educational program sessions. All participants completed the evaluation after 2 weeks after the intervention (*N* = 60).

### Data tools

#### Tool I: Precipitating factors of dry eye syndrome structured interview schedule

The researchers established tool I based on reviewing recent relevant literature [16, 19, 20] to assess the precipitating factors of DES. It is a structured interview questionnaire and is translated into the Arabic Language. This tool consists of two parts:

#### Part I: Patients’ socio-demographic characteristics

This part includes socio-demographic data of patients with DES as age, gender, marital status, level of education, place of residence, income from the perspective of the patient, and telephone number.

#### Part II: Factors associated with dry eye syndrome

This part includes precipitating factors of DES such as smoking, caffeine intake, insufficiency of vitamins such as vitamin A, vitamin D, essential fatty acids such as omega-3, wearing contact lenses, previous diagnosis of DES, using of eye lubricants or artificial tears, number of sleeping hours per day, medications, certain systemic diseases such as diabetes, rheumatoid arthritis and hypertension, previous organ transplantation, previous laser-assisted in situ keratomileuses (LASIK), and using the computer or a smartphone or other portable digital device, or watching television, electronic learning or working, and outdoor environment such as dry or windy climates.

#### Tool II: ocular surface disease index (OSDI)

This tool was developed by Allergan Inc.’s Outcomes Research Group in 1997 [21]. It was adopted by the researchers. It is a questionnaire consisting of 12 items developed to assess ocular symptoms associated with DES and their effect on functions related to vision. The researchers translated it into the Arabic language. The questionnaire is divided into three sections: ocular symptoms, functions related to vision, and environmental triggers.

### The scoring system

Patients score their answers on a scale from zero to four where zero denotes “none of the time”, one denotes “some of the time”, two denotes “half of the time”, three denotes “most of the time”; and four denotes “all of the time”. The final score is determined by multiplying the total sum of scores by 25 and subsequently dividing the result by the number of responded questions. A final score can vary from 0 to 100 with scores 0–12 means normal, 13–22 represents mild DES, 23–32 represents moderate DES, and 33–100 indicates severe DES [22].

### Data collection and analysis

#### Administrative steps

Approval of the Research Ethics Committee at the Faculty of Nursing, Alexandria University was received before initiating the study. Formal permission was received from the director of the Ophthalmology Department after explaining the study’s objectives.

#### Tool development

The researchers developed tool I, while tool II was adopted by the researchers. The tools underwent content validity testing by five experts in Medical-Surgical Nursing. Modifications were performed in response to their comments.

#### Pilot study and reliability

A pilot study involving 10% of the sample (6 patients) was carried out to assess the tools’ clarity, and applicability, and appropriate modifications were implemented. These patients were omitted from the final study sample. The tools’ reliability was measured by Cronbach’s Alpha Coefficient Test which was 0. 934 for tool I and 0.913 for tool II, representing high reliability.

#### Actual study

Participants meeting the inclusion criteria were selected from the Ophthalmology Outpatient Clinic in the previously mentioned hospital and were distributed into two equal groups: a control group which was selected first and a study group. The two groups received the usual treatment for DES including artificial tears, anti-inflammatory eye drops, and nutritional supplements such as omega-3 fatty acids supplements. The control group did not receive the educational program for DES while, the study group received the educational program for DES. Data collection commenced with the control group, followed by the study group to overcome theoretical contamination of the study group. This study was reported following the checklist for quasi-experimental studies (non-randomized experimental studies [23]. This checklist served as a guide for the study design, implementation, and reporting to guarantee methodological rigor and transparency. The actual study was executed through four phases:

**I. Assessment phase**

The researcher conducted an initial assessment of all participants in the two groups on the day of eye examination at the Ophthalmology Outpatient Clinic after diagnosis of DES. It was performed prior to the initiation of the educational nursing interventions using tool I and tool II to identify the precipitating factors of DES and to assess ocular symptoms and their effect on functions related to vision. The eye examination is performed by ophthalmologists for the diagnosis of DES using the ocular surface analyzer instrument to analyze the tear film and other specific tests such as slit lamp examination, fluorescein dye disappearance test, and Schirmer’s test.

**II. Planning phase**

The planning phase included the following activities: Identifying the required educational nursing interventions depending on the result of assessment, identifying patients’ needs, and review of related literature. It encompassed the objectives of the educational nursing interventions, the content of the educational program, teaching strategies, and learning aids. Objectives of the educational program included minimizing risk factors, reducing the severity of DE symptoms, improving visual-related functions, and preventing ocular damage. The content of the educational program included definition and causes of DES, symptoms of DES, diagnostic measures of DES, differentiation between DES and eye allergy, complications of DES, the effect of DES on vision, management of DES, self-care techniques as blink exercises and proper eyelid hygiene, proper nutrition to improve the quality of tear film, measures and precautions to prevent DES such as avoiding exposure to smoke and environmental changes, avoiding prolonged periods in air-conditioned environments; limiting contact lens use to shorter periods, avoiding staring at the computer screen or smartphone for long periods, and taking frequent breaks. In addition, health education about the importance of compliance with the treatment of DES, and instructions to maintain normal eye and vision in patients with DES (Table 1). Teaching strategies included interactive discussion. The handout booklet was designed by the researchers following reviewing recent relevant literature [24–28]. It was handed out to each participant in the study group at the first session after the assessment of DES by using tool I and tool II.

**Table 1:** Description and illustration of nursing-led educational program

Sessions	Objectives	Duration	Content
1st session	• Teach patients about the anatomy and physiology of the eye with a focus on the tear film and its components. • enhance patients’ understanding regarding DES. • Discuss causes, symptoms and risk factors of DES.	30 min	• The tear film’s structure and function. • Definition of DES • Causes and risk factors of DES such as: ♣ Environmental factors (e.g., dry or windy climates, air conditioning, extended periods of screen time) ♣ Medical condition (e.g., diabetes mellitus, autoimmune diseases) ♣ Medications that may aggravate DES (e.g., antidepressants, antihistamines) • Common symptoms of DES. • Diagnostic procedures of DES. • Differentiation between DES and eye allergy
2nd session	• Teach patients to identify early warning signs of complications such as corneal damage and infections. • Understand the effect of DES on daily activities.	30 min	• Complications of DES • The effect of DES on vision and daily activities • Management of DES (pharmacological and non-pharmacological)
3rd session	• Educate patients about several self-care techniques to manage DES, reduce the severity of DE symptoms, and improve visual-related functions. • Identify the tips of good nutrition for DES. • Emphasize to patients the value of adhering to prescribed treatment regimens and follow up visits.	30 min	• Self-care techniques: ♣ Eyelid hygiene practices (e.g., lid scrubs, warm compresses). ♣ Blink exercises. ♣ Appropriate use and application of lubricating ointments and artificial tears. • Health education about proper nutrition to improve the quality of tear film. • Importance of compliance with the recommended treatment plans for DES and follow up appointments. • Instructions to maintain normal eye and vision in patients with DES.
4th session	• Determine the lifestyle modifications needed to control DES. • Teach preventive measures to prevent exacerbation of DE symptoms.	30 min	• Lifestyle modifications: ♣ Environmental adjustments such as utilizing humidifiers. ♣ Dietary guidelines (e.g., increase omega-3 fatty acids intake). ♣ Hydration and appropriate eye protection (e.g., sunglasses or protective eye wear) • Preventive measures and precautions to prevent exacerbation of DES such as: ♣ Avoiding exposure to smoke and environmental changes ♣ Avoiding prolonged periods in air-conditioned environments ♣ Limiting contact lens use to shorter periods ♣ Avoiding staring at the computer screen or smartphone for long periods, and taking frequent breaks.

**III. Implementation phase**

The researcher established a trusting relationship through active listening to patients and answering their questions related to DES and the educational program was delivered in a private, quiet and supportive environment, where the researcher was attentive to patients’ concerns. The educational nursing interventions were individually administered to each participant in the study group in four sessions, each lasted for 30 min and held daily for four days, at the ophthalmology outpatient clinic after the assessment phase following the diagnosis of DES (Table 1). It was implemented by one researcher to ensure standardization and phone contact is maintained with patients to guarantee follow-up visits in outpatient clinics and address any queries related to the program application.

**IV. Evaluation phase**

This phase was conducted two weeks following the application of educational nursing interventions to assess the severity of DE symptoms and their effect on functions related to vision by using tool II.

### Statistical analysis

Data were entered into the computer and IBM SPSS software package version 26.0. was employed for analysis. Qualitative data was illustrated using numbers and percentages. The distribution’s normality was ensured by the Shapiro-Wilk test. Mean, standard deviation, median, and range were employed for presenting quantitative data. The results’ significance was evaluated at the 5% level. The utilized tests included the Chi-square test, Fisher’s Exact or Monte Carlo correction, Marginal Homogeneity Test, Student t-test, and Paired t-test.

## Results

Table (2) reveals that 40% of patients in the study group were in the age group from 40 to less than 50 years old while, 33.3% of the control group were in the age group from 50 to 60 years old. Most patients in the study and control groups were females (70% & 60%) and married (73.3% & 66.7%) respectively. Regarding the educational level, 30% and 33.3% of patients in the study and control groups respectively had secondary education. Furthermore, most patients in the study and control groups had insufficient income from their point of view (86.7% & 76.7%) and were living in urban areas (56.7% & 70%) respectively.

**Table 2:** Socio-demographic characteristics of patients with dry eye syndrome in the study and control groups (*n* = 60)

Socio-demographic Characteristics	Control (*n* = 30)		Study (*n* = 30)		χ^2^	*p*
	No.	%	No.	%		
Age in years						
20 < 30	6	20.0	2	6.7	3.594	^MC^p= 0.313
30 < 40	7	23.3	5	16.7		
40 < 50	7	23.3	12	40.0		
50 ≤ 60	10	33.3	11	36.7		
**Gender**						
Male	12	40.0	9	30.0	0.659	0.417
Female	18	60.0	21	70.0		
**Marital status**						
Single	6	20.0	4	13.3	1.188	^MC^p= 0.805
Married	20	66.7	22	73.3		
Divorced	2	6.7	3	10.0		
Widow	2	6.7	1	3.3		
**Level of education**						
Illiterate	7	23.3	4	13.3	3.271	0.514
Read and write	3	10.0	7	23.3		
Primary	4	13.3	6	20.0		
Secondary	10	33.3	9	30.0		
University	6	20.0	4	13.3		
**Place of residence**						
Rural	9	30.0	13	43.3	1.148	0.284
Urban	21	70.0	17	56.7		
**Income from patient point of view**						
Sufficient	7	23.3	4	13.3	1.002	0.317
Insufficient	23	76.7	26	86.7		

χ^2^: **Chi-square test** MC: **Monte Carlo**.

P: **p-value for comparing the two studied groups**.

*: **Statistically significant at ***p* ≤ **0.05**.

Table (3) shows the precipitating factors of DES. Regarding the use of prescribed drugs such as antidepressants, aspirin, and roaccutane, 53.3% of patients in both two groups were using these drugs. Regarding associated medical diseases, 43.3% of the study group had rheumatoid arthritis while an equivalent percentage (36.7%) of patients in the control group had rheumatoid arthritis and diabetes mellitus. In addition, all patients in the two groups had no history of organ transplantation. Most patients in the study and control groups had no history of LASIK surgery (66.7% & 83.3%), did not smoke (76.7% & 70%), and were not using any vitamins or nutritional supplements (80% & 63.3%) respectively. Furthermore, the majority of patients (93.3%) and (86.7%) in the study and control groups were not using vitamin D supplements. Regarding caffeine intake, 86.7% of patients in both two groups were consuming beverages containing caffeine.

**Table 3:** Precipitating factors of dry eye syndrome in the study and control groups (*n* = 60)

Precipitating Factors of Dry Eye Syndrome	Control (*n* = 30)		Study (*n* = 30)		χ^2^	*p*
	No.	%	No.	%		
Antidepressants - Aspirin – Roaccutane						
Yes	16	53.3	16	53.3	0.000	1.000
**Associated medical diseases**						
Rheumatoid arthritis	11	36.7	13	43.3	3.357	0.331
Diabetes mellitus	11	36.7	7	23.3		
Hypertension	6	20.0	4	13.3		
**Organ transplantation**						
Yes	0	0.0	0	0.0	–	–
**LASIK surgery**						
Yes	5	16.7	10	33.3	5.144	^MC^p= 0.088
**Caffeine intake**						
Yes	26	86.7	26	86.7	0.000	^FE^p= 1.000
**Smoking**						
Yes	9	30.0	7	23.3	0.341	0.559
**Vitamin and nutritional supplements**						
Yes	11	36.7	6	20.0	2.052	0.152
**Vitamin D supplements**						
Yes	4	13.3	2	6.7	0.741	^FE^p= 0.671
**Contact lenses**						
Yes	5	16.7	4	13.3	0.131	^FE^p= 1.000
**Previous diagnosis of DES**						
Yes	19	63.3	19	63.3	0.000	1.000
**Artificial tears**						
Yes	9	30.0	13	43.3	1.148	0.284
**Drinking alcohol**						
Yes	0	0.0	0	0.0	–	–
**Sleeping Hours**						
4 h	5	16.7	6	20.0	3.671	^MC^p= 0.500
5 h	7	23.3	11	36.7		
6–8 h	14	46.7	8	26.7		
9 h	2	6.7	4	13.3		
10 h	2	6.7	1	3.3		
**Smart devices (TV - Computer - Mobile)**						
1–4 h	15	50.0	15	50.0	3.162	^MC^p= 0.190
5–8 h	10	33.3	14	46.7		
> 8 h	5	16.7	1	3.3		
**Online Study or working**						
Yes	4	13.3	5	16.7	0.131	^FE^p= 1.000
Working outside						
Yes	15	50.0	12	40.0	0.606	0.436

χ^2^: **Chi-square test** MC: **Monte Carlo** FE: **Fisher Exact**.

P: **p-value for comparing the two studied groups**.

* **Statistically significant at ***p* ≤ **0.05**.

Concerning the use of contact lenses, most patients in the study and control groups had no previous history of using contact lenses (86.7% &83.3%) respectively. In addition, 63.3% of both the two groups had a history of previous DES diagnosis. 56.7% and 70.0% of the study and control groups did not use artificial tears respectively. All patients in both the two groups had a negative history of drinking alcohol. Regarding sleeping hours, 36.7% of patients in the study group sleep 5 h per day while 46.7% of the control group sleep from 6 to 8 h per day. Half of the patients (50%) in both two groups use smart devices for 1–4 h daily. Regarding the online study or work, most patients (83.3% and 86.7%) in the study and control groups respectively reported not being engaged in any online study or work, also 60% and 50% of them respectively were not working outside.

Table (4) illustrates no statistically significant difference between the two groups during the pre-test period before applying the educational program regarding all ocular symptoms, all functions related to vision, and all environmental triggers with a P-value equal to 0.126, 0.407, and 0.168 respectively. While a statistically significant difference was identified between the study and control groups two weeks after the implementation of the educational program regarding all ocular symptoms, all functions related to vision except working with a computer or ATM, and all environmental triggers with a total P-value equal to < 0.001 for all of them.

**Table 4:** Scores of ocular surface disease index (OSDI) among the study and control groups before the educational program and after 2 weeks of the intervention

Ocular Surface Disease Index (OSDI)	Control (*n* = 30)		Study (*n* = 30)		*p*_1_	*p*_2_
	Before (Mean ± SD.)	After 2 weeks (Mean ± SD.)	Before (Mean ± SD.)	After 2 weeks (Mean ± SD.)		
**Ocular Symptoms**						
Eyes sensitivity to light	2.90 ± 1.12	2.43 ± 0.97	2.80 ± 1.13	1.23 ± 0.68	0.732	< 0.001^*^
Eyes feel gritty	2.10 ± 1.54	1.03 ± 0.93	2.37 ± 1.25	0.27 ± 0.45	0.464	< 0.001^*^
Painful or sore eyes	2.87 ± 1.04	1.67 ± 0.71	3.23 ± 0.97	0.53 ± 0.51	0.164	< 0.001^*^
Blurred vision	2.57 ± 1.28	2.17 ± 1.12	2.67 ± 1.40	1.20 ± 0.76	0.773	< 0.001^*^
Poor vision	2.67 ± 1.32	2.53 ± 1.36	3.30 ± 1.29	1.73 ± 0.83	0.065	0.008^*^
**Total Score**	**13.10 ± 3.46**	**9.83 ± 2.78**	**14.37 ± 2.82**	**4.97 ± 1.83**	0.126	< 0.001^*^
t_0_ (p_0_)	12.891^*^ (< 0.001^*^)		29.357^*^ (< 0.001^*^)			
**Vision- related Functions**						
Reading	2.71 ± 1.30	2.25 ± 1.22	3.41 ± 0.97	1.41 ± 0.57	0.063	0.004^*^
Driving at night	2.40 ± 1.38	2.17 ± 1.37	2.67 ± 1.47	1.57 ± 1.01	0.472	< 0.001^*^
Working with a computer or ATM	2.65 ± 1.38	1.92 ± 0.98	2.33 ± 0.97	0.81 ± 0.51	0.374	0.390
Watching TV	2.83 ± 1.23	2.07 ± 0.91	2.73 ± 1.17	0.90 ± 0.55	0.749	0.031^*^
**Total Score**	**9.70 ± 3.95**	**7.70 ± 3.41**	**10.10 ± 3.16**	**4.30 ± 1.74**	0.407	< 0.001^*^
t_0_ (p_0_)	8.207^*^ (< 0.001^*^)		14.290^*^ (< 0.001^*^)			
**Environmental Triggers**						
Windy conditions	3.37 ± 1.0	2.97 ± 0.96	3.57 ± 0.77	2.13 ± 0.63	0.390	< 0.001^*^
Places or areas with low humidity	3.17 ± 1.26	2.90 ± 1.21	3.77 ± 0.77	2.17 ± 0.79	0.061	0.007^*^
Areas that are air conditioned	1.87 ± 1.61	1.50 ± 1.31	2.03 ± 1.71	0.73 ± 0.69	0.699	0.007^*^
**Total Score**	**8.40 ± 3.16**	**7.37 ± 2.93**	**9.37 ± 2.09**	**5.03 ± 1.38**	0.168	< 0.001^*^
t_0_ (p_0_)	6.100^*^ (< 0.001^*^)		15.424^*^ (< 0.001^*^)			

SD: **Standard deviation t: Student t-test**.

p_0_: p value for comparing between **studied periods in each group**.

p_1_: p value for comparing between **the two studied groups in pre**.

p_2_: p value for comparing between **the two studied groups in post**.

*: Statistically significant at *p* ≤ 0.05.

#: Statistically significant between **pre and post in each group**.

Table (5) reflects that the score of moderate DES in the study group significantly decreased after two weeks from implementing the educational program (values before = 36.7%, after two weeks = 3.3%). While, the score of moderate DES in the control group increased after two weeks (values before = 36.7%, after two weeks = 66.7%). In addition, the scores of severe DES were significantly decreased in both the two groups after two weeks. The table also shows no statistically significant difference between the two groups regarding the overall score of OSDI before implementing the educational program (*P* = 0.222) while there was a statistically significant difference two weeks after implementing the educational program (P = < 0.001).

**Table 5:** Overall scores of ocular surface disease index (OSDI) among the study and control groups before the educational program and after 2 weeks of the intervention

Ocular Surface Disease Index (OSDI)	Control (*n* = 30)				Study (*n* = 30)				Test of Sig. (*p*_1_)	Test of Sig. (*p*_2_)
	Before		After 2 weeks		Before		After 2 weeks			
	No.	%	No.	%	No.	%	No.	%		
Normal (0–12)	0	0.0	1	3.3	0	0.0	11	36.7	χ^2^ = 3.007 (^MC^p= 0.222)	χ^2^ = 32.993^*^ (^MC^p <0.001^*^)
Mild dry eye disease (13–22)	5	16.7	8	26.7	1	3.3	18	60.0		
Moderate dry eye disease (23–32)	11	36.7	20	66.7	11	36.7	1	3.3		
Severe dry eye disease (≥ 33)	14	46.7	1	3.3	18	60.0	0	0.0		
MH (p)	57.0^*^ (< 0.001^*^)				78.500^*^ (< 0.001^*^)					
Total Score (0–48)										
Min. – Max.	16.0–41.0		11.0–35.0		21.0–42.0		9.0–26.0		t = 1.704 (0.094)	t = 7.941^*^ (< 0.001^*^)
Mean ± SD.	31.20 ± 7.20		24.90 ± 5.99		33.83 ± 5.24		14.30 ± 3.71			
Median	32.0		26.0		34.0		13.50			
% Score										
Min. – Max.	36.36–85.42		25.0–72.92		43.75–90.91		18.75–59.09			
Mean ± SD.	67.02 ± 15.66		53.43 ± 12.82		73.11 ± 11.76		30.98 ± 8.68			
Median	71.67		56.53		73.96		29.36			
t_0_ (p)	13.474^*^ (< 0.001^*^)				28.879^*^ (< 0.001^*^)					

SD: **Standard deviation** t: **Student t-test** t_0_: **Paired t-test**.

χ^2^: **Chi-square test**.

MC: **Monte Carlo**.

MH: **Marginal Homogeneity Test**.

P: **p-value for comparing the studied periods in each group**.

• P_1_: **p-value for comparing the two studied groups in pre**.

• P_2_: **p-value for comparing the two studied groups in post**.

*: **Statistically significant at ***p* ≤ **0.05**.

## Discussion

Dry eye syndrome is a multifactorial disorder which is prevalent and frequently diagnosed in ophthalmology that was reported to limit the patients’ ability to perform daily activities, hinder work productivity, and affect their quality of life [29]. The present study was conducted to determine the impact of an educational program on health-related outcomes of patients with DES. Findings support that patients who received the nursing-led educational program experienced less severity of DE symptoms and less influence of DE symptoms on visual-related functions compared to patients who did not receive the program.

Regarding socio-demographic findings, the results revealed that the majority of the studied participants were females, married, living in urban areas and their ages varied between 40 and 60 years old. This is congruent with (Osae et al., 2020) [30] who found most participated patients with DES were females and (Shanti et al., 2020) [16] who reported that the average age of all subjects was 43.61 ± 18.57 years. That gender effect falls in the domain of the hormonal differences that affect the ocular structure, functioning, and health from both molecular levels expressed in the form of protein synthesis, tissue morphology, and gene, as well as, physiological level in the form of aqueous tear output and tear film stability in women [31].

Concerning the precipitating factors of DES, more than half of the patients in both the study and control group were taking antidepressants, aspirin, and roaccutane, and almost half of them had associated medical diseases as rheumatoid arthritis and diabetes mellitus. Medications having anticholinergic properties cause deficiency in aqueous and mucin layers in the tear film leading to DES [15]. Similarly, (Jones et al., 2017) and (Choi et al., 2018) [32, 33] emphasized that medications reported to exacerbate DES include antihistamines, β-blockers, congestion relievers, diuretic drugs, tranquilizers, tricyclic antidepressants and antipsychotic drugs, oral contraceptive pills, estrogen replacement therapy, and anti-Parkinson’s drugs.

In addition, the results supported that about a quarter of the study’s participants suffered from diabetes mellitus. As per the American Diabetes Association (2023), 54% of diabetic patients are suffering from DES [34]. Pathologically- high blood glucose levels, insufficient insulin levels, and compromised immunity among diabetic patients impair the functions of the lacrimal glands. The affliction manifests as poor quantity/quality of tears, decreased tear production, and adhesion to the eye [35]. It goes hand in hand with (Tat et al., 2024) [36] who reported that the prevalence of dry eyes in DM patients also ranges from 15 to 53%. The pathogenesis of dry eye caused by diabetes is mainly related to peripheral corneal neuropathy, tear film instability, ocular surface inflammation, and the apoptosis of conjunctival epithelial cells. Another mechanism involved is that with prolonged hyperglycemia, tear osmolarity increases, while conjunctival mucus secretion is significantly reduced, leading to decreased tear secretion and increased tear film instability.

Moreover, one-third of patients in the study group and less than one-quarter of the control group had a positive history of previous LASIK Surgery. The exposure of the ocular surface to surgical changes affects the blinking pattern causing alteration in the flow of the tears and affecting its stability [37]. Furthermore, it was identified that most patients in both groups were females, had a history of DES, and did not use artificial tears. On the same hand, (Mohammed et al., 2022) [29] reported a significant association between the usage of eye drops, previous history of DES, female gender, and the prevalence of DES.

The current study also reflected that the majority of participants were not using any vitamins or nutritional supplements, not using vitamin D supplements, and were caffeine consumers. Moreover, nearly half of them had inadequate sleeping hours. This is congruent with results reported by (Abu-Ismail et al., 2023) [38] who found that among Jordanian medical students; female gender, caffeine consumption, and poor quality of sleep are linked to elevated OSDI scores, which reveals that it might be taken into consideration as contributing factors for DES. Sailing on the same boat as (Molina-Leyva et al., 2017) [39] found that elevated use of omega-3 supplements enhances tear formation and secretion, and relieves symptoms of DES.

Moreover, the results revealed that all patients were using smart devices for prolonged periods. Maintaining prolonged eye contact with the screen without adequate blinking may contribute to DES. In a Korean study, (Choi et al., 2018) [33] found that dry eye symptoms had considerably greater rates for women, persons who use contact lenses, and users of a computer and/or smartphone for longer than three hours a day. In line with (Beyoglu et al., 2021) [40] stated that online education during the COVID-19 pandemic, television, computer, and tablet usage had resulted in significant changes in OSDI scores and dry eye symptoms. Similarly, (García-Ayuso et al., 2022) [41] found that female gender, contact lens wear, and online class attendance were linked to a higher incidence of DE symptoms.

A noteworthy finding of the current study is that the educational program had affected positively patients with DES, as the experimental group had experienced a significant health-related outcomes improvement in terms of a statistically significant difference overall score of OSDI between the control and study group 2 weeks after applying the educational program. Moreover, the score of moderate DES in the study group significantly decreased two weeks after applying the educational program. Regarding ocular symptoms, functions related to vision, and environmental triggers, a statistically significant difference between the two groups after two weeks of implementing the educational program was found in favor of the study group. This goes hand in hand with (Lee, 2015) [42] who found that significant increase in the mean score after applying the educational program for patients with DES. On the same hand, (Sano, 2018) [43] emphasized that health education promoting ocular surface protection methods should be considered in the management plan of patients with DES.

## Conclusion

This study concludes that the educational interventions induced significant changes as patients who received the educational program experienced less severity of dry eye symptoms and less effect of DES on visual-related functioning than those who did not receive it. It is necessary to raise awareness and understanding among patients, medical professionals, paramedical staff, and general ophthalmologists about the benefits of including the educational program in the management protocol of patients with DES.

### Recommendations

The educational program should be included in the nursing care for patients with DES to help them understand the condition, actively participate in their care, manage symptoms effectively, and maintain eye health. The developed booklet with colorful illustrations, information, and guidance on self-care practices must be given to each patient with DES in the outpatient clinics. It is suggested to repeat the study using a large probability sample.

### Limitations

The long-term effect of the educational program on health outcomes needs to be investigated as the study illustrated the effect only after 2 weeks from implementation of the program. In addition, lack of randomization, and the study was conducted at a single Ophthalmology Outpatient Clinic, which may affect the generalizability of the findings.

## Data Availability

The datasets used and/or analysed during the current study are available from the corresponding author on reasonable request.

## Abbreviations

DE: Dry Eye
DES: Dry Eye Syndrome
OSDI: Ocular Surface Disease Index
LASIK: Laser-Assisted in Situ Keratomileuses

## References

[1] Al-Mohtaseb Z, Schachter S, Lee BS, Garlich J, Trattler W. The relationship between dry eye disease and digital screen use. Clin Ophthalmol. 2021;15:3811–20. 10.2147/OPTH.S321591. 34531649 10.2147/OPTH.S321591 PMC8439964

[2] Akpek EK, Amescua G, Farid M, Garcia-Ferrer FJ, Lin A, Rhee MK, Varu DM, Musch DC, Dunn SP, Mah FS, American Academy of Ophthalmology Preferred Practice Pattern Cornea and External Disease Panel. Dry eye syndrome preferred practice pattern^®^. Ophthalmology. 2019;126(1):P286–334. 10.1016/j.ophtha.2018.10.023. 30366798 10.1016/j.ophtha.2018.10.023

[3] Shtein RM, Jacobs DS, Givens J. Dry eye disease. 2024. https://www.uptodate.com/contents/dry-eye-disease. Accessed 6 Jan 2024.

[4] Rouen PA, White ML. Dry eye disease. Home Healthc Now. 2018;36(2):74–83. 10.1097/NHH.0000000000000652. 29498987 10.1097/NHH.0000000000000652

[5] Kiziltan ME, Dogan C, Ayas S, Valls-Sole J, Gunduz A. Changes in brainstem excitatory and inhibitory pathways in dry eye syndrome. Neurosci Lett. 2020;718:134726. 10.1016/j.neulet.2019.134726. 31884018 10.1016/j.neulet.2019.134726

[6] Kopacz D, Niezgoda L, Fudalej E, Nowak A, Maciejewicz P. Tear film–physiology and disturbances in various diseases and disorders. Ocular Surface Diseases—Some Current Date on Tear Film Problem and Keratoconic Diagnosis. IntechOpen. 2020:137 – 44. 10.5772/intechopen.94142

[7] Jeon J, Park S. Comparison of the efficacy of eyelid warming masks and artificial tears for dry eye symptoms in contact lens wearers. Contact Lens Anterior Eye. 2021;44(1):30–4. 10.1016/j.clae.2020.02.013. 32169321 10.1016/j.clae.2020.02.013

[8] Phadatare SP, Momin M, Nighojkar P, Askarkar S, Singh K. A comprehensive review on dry eye disease: diagnosis, medical management, recent developments, and future challenges. Adv Pharm. 2015;2015:1–12. 10.1155/2015/704946.

[9] Yoo TK, Oh E. Diabetes mellitus is associated with dry eye syndrome: a meta-analysis. Int Ophthalmol. 2019;39(11):2611–20. 10.1007/s10792-019-01110-y. 31065905 10.1007/s10792-019-01110-y

[10] Kojima T, Dogru M, Kawashima M, Nakamura S, Tsubota K. Advances in the diagnosis and treatment of dry eye. Prog Retin Eye Res. 2020;78:100842. 10.1016/j.preteyeres.2020.100842. 10.1016/j.preteyeres.2020.100842 32004729

[11] Golden MI, Meyer JJ, Patel BC. Dry eye syndrome. In: StatPearls [Internet]. Treasure Island (FL): StatPearls Publishing; 2024. https://www.ncbi.nlm.nih.gov/books/NBK470411/. Accessed 10 Jan 2024. 29262012

[12] Şimşek C, Doğru M, Kojima T, Tsubota K. Current management and treatment of dry eye disease. Turkish J Ophthalmol. 2018;48(6):309–13. 10.4274/tjo.69320. 10.4274/tjo.69320 PMC6330664 30605938

[13] Abdulmannan DM, Naser AY, Ibrahim Ok, Mahmoud AS. Visual health and prevalence of dry eye syndrome among university students in Iraq and Jordan. BMC Ophthalmol. 2022;22(1):265. 10.1186/s12886-022-02485-w. 35698109 10.1186/s12886-022-02485-w PMC9192247

[14] Wu Y, Wang C, Wang X, Mou Y, Yuan K, Huang X, Jin. X. advances in dry eye disease examination techniques. Front Med. 2022;8:826530. 10.3389/fmed.2021.826530. 10.3389/fmed.2021.826530 PMC8823697 35145982

[15] Donthineni PR, Shanbhag SS, Basu S. An evidence-based strategic approach to prevention and treatment of dry eye disease, a modern global epidemic. Healthcare. 2021;9(1):89. 10.3390/healthcare9010089. 33477386 10.3390/healthcare9010089 PMC7830429

[16] Shanti Y, Shehada R, Bakkar MM, Qaddumi J. Prevalence and associated risk factors of dry eye disease in 16 northern West Bank towns in Palestine: a cross-sectional study. BMC Ophthalmol. 2020;20(1):1–8. 10.1186/s12886-019-1290-z. 31931756 10.1186/s12886-019-1290-z PMC6958733

[17] Kuo YK, Lin IC, Chien LN, Lin TY, How YT, Chen KH, Dusting GJ, Tseng CL. Dry eye disease: a review of epidemiology in Taiwan, and its clinical treatment and merits. J Clin Med. 2019;8(8):1227. 10.3390/jcm8081227. 31443274 10.3390/jcm8081227 PMC6722537

[18] Sim J, Lewis M. The size of a pilot study for a clinical trial should be calculated in relation to considerations of precision and efficiency. J Clin Epidemiol. 2012;65(3):301–8. 10.1016/j.jclinepi.2011.07.011. 22169081 10.1016/j.jclinepi.2011.07.011

[19] Qian L, Wei W. Identified risk factors for dry eye syndrome: a systematic review and meta-analysis. PLoS ONE. 2022;17(8):e0271267. 10.1371/journal.pone.0271267. 35984830 10.1371/journal.pone.0271267 PMC9390932

[20] Wolffsohn JS, Wang MTM, Vidal-Rohr M, Menduni F, Dhallu S, Ipek T, Acar D, Recchioni A, France A, Kingsnorth A, Craig JP. Demographic and lifestyle risk factors of dry eye disease subtypes: a cross-sectional study. Ocul Surf. 2021;21:58–63. 10.1016/j.jtos.2021.05.001. 33965652 10.1016/j.jtos.2021.05.001

[21] Walt JG, Rowe MM, Stern KL. Evaluating the functional impact of dry eye: the ocular surface Disease Index. Drug Inform J. 1997;31:1436.

[22] Grubbs JR, Tolleson-Rinehart S, Huynh K, Davis RM. A review of quality of life measures in dry eye questionnaires. Cornea. 2014;33(2):215–8. 10.1097/ICO.0000000000000038. 24326332 10.1097/ICO.0000000000000038 PMC4201928

[23] Tufanaru C, Munn Z, Stephenson M, Aromataris E. Checklist for quasi-experimental studies (non-randomized experimental studies). Adelaide: Joanna Briggs Institute; 2017. https://jbi.global/sites/default/files/2020-08/Checklist_for_Quasi Experimental_Appraisal_Tool.pdf. Accessed 20 Aug 2024.

[24] Duffy MA. Eye Health: Anatomy of the Eye. 2021. https://visionaware.org/your-eye-condition/eye-health/anatomy-of-the-eye/. Accessed 5 Dec 2022.

[25] Mukamal R. Facts about Tears. American Academy of Ophthalmology. 2021. https://www.aao.org/eye-health/tips-prevention/facts-about-tears. Accessed 8 Dec 2022.

[26] O’Neil EC, Henderson M, Massaro-Giordano M, Bunya VY. Advances in dry eye disease treatment. Curr Opin Ophthalmol. 2019;30(3):166–78. 10.1097/ICU.0000000000000569. 30883442 10.1097/ICU.0000000000000569 PMC6986373

[27] Weiner G. Dry eye disease. American Academy of Ophthalmology, Eye Net Magazine. 2018; 43–48. https://www.aao.org/eyenet/article/dry-eye-disease. Accessed 9 Dec 2022.

[28] American Optometric Association. Dry eye. 2022. https://www.aoa.org/healthy-eyes/eye-and-vision-conditions/dry-eye?sso=y. Accessed 9 Dec 2022.

[29] Mohammed S, Kefyalew B, Kebede BN, Lorato M. Prevalence and associated factors of symptomatic dry eye among undergraduate students in Hawassa University College of Medicine and Health Sciences, Hawassa. Ethiopia BMJ Open Ophthalmol. 2022;7:e001149. 10.1136/bmjophth-2022-001149.

[30] Osae EA, Ablordeppey RK, Horstmann J, Kumah DB, Steven P. Clinical Dry Eye and Meibomian Gland features among Dry Eye patients in Rural and Urban Ghana. Clin Ophthalmol. 2020;14:4055–63. 10.2147/OPTH.S275584. 33262570 10.2147/OPTH.S275584 PMC7699986

[31] Matossian C, McDonald M, Donaldson KE, Nichols KK, MacIver S, Gupta PK. Dry eye disease: consideration for women’s health. J Women’s Health. 2019;28(4):502–14. 10.1089/jwh.2018.7041. 10.1089/jwh.2018.7041 PMC6482917 30694724

[32] Jones L, Downie LE, Korb D, Benitez-del-Castillo JM, Dana R, Deng SX, Craig JP. TFOS DEWS II management and therapy report. Ocul Surf. 2017;15(3):575–628. 10.1016/j.jtos.2017.05.006. 28736343 10.1016/j.jtos.2017.05.006

[33] Choi J, Kim K, Kim H, Joo S, Cha H. Factors influencing on dry eye symptoms of university students using smartphones. Indian J Public Health Res Dev. 2018;9(11):964. 10.5958/0976-5506.2018.01583.8.

[34] American Diabetes Association. Eye health: dry eye with diabetes. 2023. https://diabetes.org/sites/default/files/2023-09/EyeHealth_Resource_Dry-Eye_rev-1.pdf. Accessed 20 Aug 2024.

[35] Zhang X, Zhao L, Deng S, Sun X, Wang N. Dry eye syndrome in patients with diabetes mellitus: prevalence, etiology, and clinical characteristics. J Ophthalmol. 2016;2016(1):8201053. 10.1155/2016/8201053. 27213053 10.1155/2016/8201053 PMC4861815

[36] Tat TT, Duc KN, Hong PP, Sa HN, Trung KN, Thu HN, Ha KL, Huu DN, Doan TT, Viet TL. Dry eye and some related factors in patients with type 2 diabetic nephropathy: a cross-sectional study in Vietnam. Clin Ophthalmol. 2024;18:1217–24. 10.2147/OPTH.S458633. 38737597 10.2147/OPTH.S458633 PMC11088030

[37] Chen Q, Li M, Yuan Y, Me R, Yu Y, Shi G, Wang X, Ke B. Effects of tear film lipid layer thickness and blinking pattern on tear film instability after corneal refractive surgery. Cornea. 2017;36(7):810–5. 10.1097/ICO.0000000000001207. 28410354 10.1097/ICO.0000000000001207

[38] Abu-Ismail L, Abuawwad M, Taha M, Khamees A, Abu Ismail D, Sanwar M, Al-Bustanji Y, Nashwan A, Alameri O, Alrawashdeh H, Serhan H, Abu-Ismail J. Prevalence of Dry Eye Disease among Medical students and its Association with Sleep habits, Use of Electronic devices and Caffeine Consumption: a cross-sectional questionnaire. Clin Ophthalmol. 2023;17:1013–23. 10.2147/OPTH.S397022. 37035514 10.2147/OPTH.S397022 PMC10081668

[39] Molina-Leyva I, Molina-Leyva A, Bueno-Cavanillas A. Efficacy of nutritional supplementation with omega-3 and omega-6 fatty acids in dry eye syndrome: a systematic review of randomized clinical trials. Acta Ophthalmol. 2017;95(8):e677–85. 10.1111/aos.13428. 28371493 10.1111/aos.13428

[40] Beyoglu A, Beyoglu MM. The effect of online education during the pandemic on ocular surface symptoms. J Surg Med. 2021;5(9):928–31. 10.28982/josam.989477.

[41] García-Ayuso D, Di Pierdomenico J, Moya-Rodríguez E, Valiente-Soriano FJ, Galindo-Romero C, Sobrado-Calvo P. Assessment of dry eye symptoms among university students during the COVID-19 pandemic. Clin Experimental Optometry. 2022;105(5):507–13. 10.1080/08164622.2021.1945411. 10.1080/08164622.2021.1945411 34279190

[42] Lee SR. Adoption of an educational program for the prevention of dry eye syndrome among adults in urban areas. Int Inform Inst (Tokyo) Inform. 2015;18(9):4051–56.

[43] Sano K, Kawashima M, Takechi S, Mimura M, Tsubota K. Exercise program improved subjective dry eye symptoms for office workers. Clin Ophthalmol. 2018;12:307–11. 10.2147/OPTH.S149986. 29445264 10.2147/OPTH.S149986 PMC5810522

